# Prediction of Tumor Mutation Load in Colorectal Cancer Histopathological Images Based on Deep Learning

**DOI:** 10.3389/fonc.2022.906888

**Published:** 2022-05-24

**Authors:** Yongguang Liu, Kaimei Huang, Yachao Yang, Yan Wu, Wei Gao

**Affiliations:** ^1^ Department of Anorectal Surgery, Weifang People’s Hospital, Weifang, China; ^2^ Genies (Beijing) Co., Ltd., Beijing, China; ^3^ Qingdao Geneis Institute of Big Data Mining and Precision Medicine, Qingdao, China; ^4^ School of Mathematics and Statistics, Hainan Normal University, Haikou, China; ^5^ Department of Internal Medicine-Oncology, Fujian Cancer Hospital and Fujian Medical University Cancer Hospital, Fuzhou, China

**Keywords:** immunotherapy, colorectal cancer, deep learning, tumor mutational burden, ResNet

## Abstract

Colorectal cancer (CRC) is one of the most prevalent malignancies, and immunotherapy can be applied to CRC patients of all ages, while its efficacy is uncertain. Tumor mutational burden (TMB) is important for predicting the effect of immunotherapy. Currently, whole-exome sequencing (WES) is a standard method to measure TMB, but it is costly and inefficient. Therefore, it is urgent to explore a method to assess TMB without WES to improve immunotherapy outcomes. In this study, we propose a deep learning method, DeepHE, based on the Residual Network (ResNet) model. On images of tissue, DeepHE can efficiently identify and analyze characteristics of tumor cells in CRC to predict the TMB. In our study, we used ×40 magnification images and grouped them by patients followed by thresholding at the 10th and 20th quantiles, which significantly improves the performance. Also, our model is superior compared with multiple models. In summary, deep learning methods can explore the association between histopathological images and genetic mutations, which will contribute to the precise treatment of CRC patients.

## Introduction

Colorectal cancer (CRC), including colon cancer and rectal cancer, is one of the top 3 malignant tumors in the world for morbidity and mortality (1–3). According to statistics from the American Cancer Society, the estimated death toll in 2021 even reached 149,500 (4). In China, 25% of patients experience metastasis during diagnosis or treatment, and the 5-year survival rate of patients is less than 5% (5). The treatment of CRC is mostly based on surgery and chemotherapy. However, because tumor cells grow rapidly and are prone to metastasis, surgery and chemotherapy can only temporarily relieve the disease but cannot completely cure it. Immunotherapy kills tumors by activating the host immune system that is anti-deteriorating and durable; this has become the focus of the cancer treatment field in the new era.

In tumor immunotherapy, programmed death receptor programmed cell death-1/programmed cell death-ligand 1 (PD-1/PD-L1) inhibitors and cytotoxic T lymphocyte-associated antigen 4 (CTLA-4) inhibitors are the main immune checkpoint inhibitors (ICIs) (6, 7). Several clinical studies have proven that compared with platinum-based chemotherapy and surgery, immunotherapy can improve the overall survival rate of patients in most cancers (8–14). However, not all patients respond well in the clinical treatment with ICIs. Several studies have found that the efficacy of ICIs is closely related to the level of PD-L1 (15). So mastering the immune microenvironmental response of patients is a critical requirement. Previously, PD-L1 expression was the main marker for predicting the effect of immunotherapy. Several solid tumor studies have shown the effectiveness of PD-L1 expression detection, such as melanoma, CRC, and non-small cell lung cancer (NSCLC) (16, 17). However, as immunotherapy research continues to progress, the insufficient detection of PD-L1 expression has shown that it is no longer the only criterion to predict the efficacy of ICIs (18). In this regard, tumor mutational burden (TMB) appears as a marker of ICI efficacy identification and plays an irreplaceable role in optimizing targeted regimens and developing well-tolerated drugs for physicians.

The definition of TMB is the number of mutations per megabase in the coding region of the tumor exome. With an increase of the TMB, the degree of acquired somatic mutations will increase and more tumor-specific neoantigens will be released. A portion of the antigen is presented on the human leukocyte antigen (HLA) molecules on the surface of the cancer cells, thus triggering the recognition and processing of the tumor cells by the immune system. The reflection of TMB on PD-L1 levels affects the formulation of ICI regimens for patients in the clinic, which has aroused great interest in the determination of the TMB in tumors by researchers in various fields. According to current research, there is almost no correlation between TMB and PD-L1 expression in many cancers and their subtypes, including NSCLC, CRC, melanoma, and pancreatic cancer, which indicates that the TMB can serve as an independent prognostic marker (19, 20). Cao et al. (21) compiled the survival indicators of 103,078 patients with different cancers and included 45 immune-related studies; they finally found that TMB-H (high tumor mutational burden) patients achieved better survival after receiving immunotherapy. In 2020, the Food and Drug Administration (FDA) for first time approved TMB to be used as a diagnostic marker for pan-cancer immunotherapy when unresectability or metastasis occurs (22). In general, TMB-H has predictive and prognostic potential for the immunotherapy of solid tumors.

Although many studies have proven that the TMB performs well as a marker in ICI treatment, it is still hard to accurately measure and define the threshold of TMB-H (23). At present, whole-exome sequencing (WES) is the main method of TMB quantification that quantifies the TMB directly and comprehensively. WES data sets are often used in tumors to show the correlation between ICI reaction and TMB status (24, 25). Although this method can measure the TMB with high standards, it has some stringent requirements. For example, it not only requires fresh and high-quality samples but also is expensive and has a long working time (26). Therefore, low-cost targeted sequencing panel detection is often used as an alternative measurement to WES; it infers full mutation burden from a narrower sequencing space, leading to the development of an integrated MSK-IMPACT assay by Zehir et al. The assay can evenly cover clinically relevant genes and fusions of target genes, so the TMB content can be accurately estimated (27). However, targeted sequencing has some fatal drawbacks. Buchhalter et al. (28) evaluated the Illumina TSO500 panel and found that the 1.5–3-Mbp panel is more suitable for TMB estimation, and lower ranges will bring errors in TMB estimation, while targeted sequencing cannot detect small sequencing ranges and only targets tumor cells with repeated mutations. Deep learning has shown an ultrahigh level in processing complex and large amounts of information in histopathological images. Deep convolutional neural networks (CNN) have yielded many shocking research results in image feature recognition of cancer histopathology (29, 30). Moreover, Mika et al. developed the Image2TMB method with deep learning to measure the TMB in lung adenocarcinoma pathological tissue images at three scales (×5, ×10, and ×20 magnification) (31). Finally, the performance of the ×20 scale is the best with an area under the curve (AUC) of 0.81, showing that high-resolution scale images are more conducive to the prediction of markers. Therefore, there is indeed a correlation between the tumor somatic mutations and gene mutations, and deep learning can evaluate this correlation well. To further understand the capability of deep learning for tumor cell somatic recognition in histopathological images and to explore efficient strategies for TMB measurement, this paper builds a deep learning model, DeepHE. This can automatically analyze the TMB in pathological images and predict the probability of their potential TMB from CRC whole slices [whole-section images (WSIs)] in The Cancer Genome Atlas (TCGA). We downloaded the entire formalin-fixed paraffin-embedded (FFPE) tissue data of CRC at ×40 resolution and grouped them by patients. To train a more efficient model, a higher resolution compared to that of previous studies is chosen. Also, the classification by patients avoids errors caused by the same pathological tissue images from one patient. The images of 509 patients remained after DeepHE sorted and filtered data by patients, then we segmented the WSI slice and performed color normalization. To improve its feature recognition ability and speed up the convergence, this research introduced a residual network model derived from CNN technology and then trained the model of ResNet50 by 2-fold cross-validation. The performance of the five models including ResNet18, ResNet34, VGG16, AlexNet, and ResNet50 showed superiority. This study provides an important way for patients to benefit from ICI treatment and explores the relationship between the TMB and the tumor immune microenvironment.

## Results

### The Workflow of DeepHE

**Figure 1:**
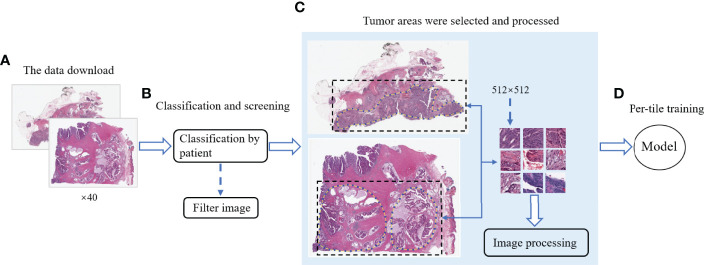
The workflow of this study. **(A)** Download the CRC image data of ×40 resolution from TCGA. **(B)** Categorize the data by patients and remove the unqualified images. **(C)** Mark the tumor area and segment it, and then perform noise removal and color normalization processing. **(D)** Model training and testing.

**Figure 1** shows the workflow of DeepHE. The ×40 scale images contain more details, and our model still performs well in predicting the TMB on CRC in a short time. From 611 patients, a total of 509 patients were left by professional pathologists, of which 12 patients were removed because they lacked TMB information or the tumor area could not be annotated. The clinical information of the remaining 509 CRC patients was also obtained and collated from TCGA (**Table 1**). Then 1,586,826 qualified slices were derived from the images of these patients, which are approximately 3,117 slices in each group. After that, each group was randomly divided into two groups as the training set and validation set, resulting in two sets of 793,413 H&E slices for model training and testing.

**Table 1:** Clinical Information for TCGA Colorectal Cancer Patients.

Clinical variable	Category	Number of patients
Tumor stage	Stage I	88
	Stage II	178
	Stage III	147
	Stage IV	75
	Unknown	21
Prior malignancy	Yes	55
	No	451
	Unknown	2
AJCC pathologic T	T1	15
	T2	91
	T3	341
	T4	58
	Unknown	4
AJCC pathologic N	N0	282
	N1	130
	N2	91
	Nx	2
	Unknown	4
AJCC pathologic M	M0	366
	M1	74
	Mx	58
	Unknown	10
Gender	Women1	246
	Men0	260
	Unknown	3
Vital status	Alive	395
	Dead	111
	Unknown	3
Age at index	≥66	272
	<66	237
New tumor event after initial treatment	Yes	91
	No	332
	Unknown	86

### DeepHE Achieves Relatively Good Performance in the Tumor Mutational Burden Prediction

According to the standard of dividing the TMB level in a previous related work, this study uses the thresholds of 10 and 20 (32). Then, the research divided the data into TMB-H (TMB high) and TMB-L (TMB low), satisfying H:L (10) = 83:426 (with a threshold of 10) and H:L (20) = 73:426 (with a threshold of 20), after which the model was trained and tested on the FFPE dataset. When the data set used a TMB threshold of 10, the ratio of the number of patients between TMB-H and TMB-L is 83:426.

**Figure 2:**
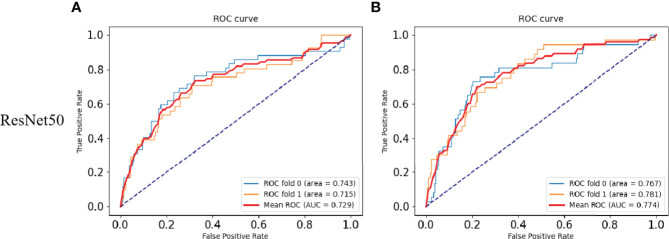
Results of the TMB prediction model. **(A)** ROC plot of the ResNet50 model with a TMB cutoff of 10 under 2-fold cross-validation. **(B)** ROC plot of the ResNet50 model with a TMB cutoff of 20 under 2-fold cross-validation.

The performances of DeepHE in predicting TMB at different thresholds were shown in **Figure 2**. ResNet50 with more hidden layers in the residual network is selected to capture a more detailed feature information. The results also showed that the AUC of ResNet50 reached 0.729 under 2-fold cross-validation. Then, when the threshold is 20, H:L (20) = 73:436, the AUC of ResNet50 is 0.774. During the whole trial, 30 epochs were maintained, which was the best value obtained after many attempts. At the beginning of the experiment, the epoch was set to 50. However, as the epoch was set greater than 30, the training ACC result has stabilized at around 0.9679, which means that a larger value will only take more time.

### DeepHE Achieves Higher Areas Under the Curve Than Those of Existing Methods in the Tumor Mutational Burden Prediction

**Figure 3:**
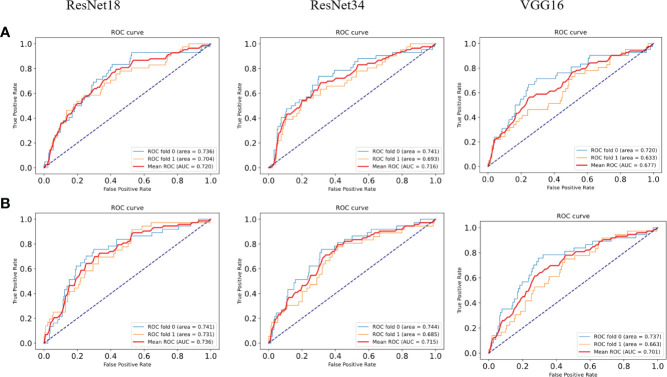
ROC curves of the comparison models. **(A)** ROC plots for different models with a TMB cutoff of 10 under 2-fold cross-validation. **(B)** ROC plots for different models with a TMB cutoff of 20 under 2-fold cross-validation.

To verify the superiority of the model performance, we tested four models that have contributed greatly to the current image recognition field based on the same process, namely, ResNet18, ResNet34, VGG16, and AlexNet. The comparisons between their results and our model are shown in **Figure 3**. We used a sliding window to visualize the probability value on each small slice, classified and counted the TMB level on the slice contained in each complete WSI. When the ratio of the number of slices containing TMB-H in a patient to the number of all his eligible slices is greater than 50%, the patient was identified as TMB-H and *vice versa* for TMB-L. It is worth noting that the eligibility here refers to slices owned by the patient that were not screened out and participated in the training. **Figure 3A** shows the ROC curves of the TMB divided by 10 into all methods, and **Figure 3B** shows the case where 20 is the threshold. After training of ResNet18, the AUC is 0.720; at H:L (20), the AUC is 0.736. It can be found that the results of ResNet18 are very close to that of ResNet50, but there are still some gaps. We suspect that as the number of layers increased, the results would get closer to our model or even surpass it. As a result, we experimented with ResNet34. When the threshold is 10, the AUC value of the ROC curve is 0.716, and when H:L (20) = 73:426, the AUC is 0.715. Next, we tested VGG16 and AlexNet. When AlexNet was split at 20, the AUC only reached 0.685, so it was not tested with a threshold of 10. In the study, VGG16 made the TMB at H:L (10) = 83:426, the AUC value of the ROC curve was 0.677, H:L (20) = 73:426, and the AUC value was 0.701. The reason is that compared with AlexNet, VGG16 has more convolutional layers and smaller pooling kernels, which can extract more detailed information, but the model requires more parameters to participate, which will occupy more or a large memory space (33). Taken together, the results are that ResNet50 performs well, while AlexNet performs the least well. For ResNet, VGG, and AlexNet models, AlexNet has the least number of convolutional layers, which may be one of the reasons for its worst effect.

At the same threshold, the performances of all models are comparable. We perform statistics on performance metrics of all models used in the study in **Tables 2**, **3**, namely, accuracy (ACC), precision, recall, and F1 score. After statistics highlighting the optimal value in red, it was found that DeepHE based on ResNet50 has always maintained a relatively high performance.

**Table 2:** Comparison of the performance of different models (TMB cutoff = 10).

Model	ACC	Precision	Recall	F1-score
ResNet18	0.820	0.640	0.562	0.575
ResNet34	0.815	0.607	0.548	0.552
ResNet50	0.830	0.681	0.587	0.605
VGG16	0.830	0.681	0.587	0.605

**Table 3:** Comparison of performance of different models (TMB cutoff = 20).

Model	ACC	Precision	Recall	F1-score
ResNet18	0.835	0.622	0.564	0.575
ResNet34	0.845	0.649	0.548	0.555
ResNet50	0.850	0.677	0.567	0.582
VGG16	0.845	0.620	0.530	0.530
AlexNet	0.840	0.643	0.563	0.575

## Discussion

TMB has emerged as a biomarker responsive to the efficacy of immunotherapy and has been approved by the FDA. In 2018, Gandara et al. (34) for first time demonstrated that the TMB can stably predict the effect of immunotherapy. In this study, the content of the TMB was determined on pathological images of CRC. For the selection of the threshold, the researchers found that in patients with NSCLC, when the TMB ≥10 mut/Mb was used as the cutoff point, it was found that patients with high TMB content were more responsive to immunotherapy. ICI treatment prolonged the progression-free survival rate of these patients and far exceeded the effect of platinum-based doublet chemotherapy (35). Therefore, the ResNet50-based DeepHE had a threshold of 10 and 20, respectively. When the threshold was 10, the area under the ROC curve reached 0.729. At the same time, when the threshold was 20, the AUC of ResNet50 was 0.774. Ciardiello et al. (36) studied colon cancer with mismatch repair deficiency (dMMR), and high microsatellite instability (MSI-H) concluded that immunotherapy has a certain clinical therapeutic effect. The TMB has been reported as an important marker for concomitant CRC immunotherapy, which fully justifies our trial design. Furthermore, the addition of residual blocks further improves the performance of the model. Therefore, our research is bound to have an important impact on improving the survival rate of cancer patients. The detection of TMB mainly relies on WES technology, which is expensive, requires a lot of time (about 60 days), will delay the treatment time of cancer patients, and is likely to make the best treatment time missed. In contrast, the DeepHE method does not require a large amount of biopsy tissue sample, a lot of manpower, material resources, and time. It only needs to run the trained model on the pathological images of patients.

In recent years, machine learning methods have been widely used in biomedical research like drug repositioning (37, 38) and single-cell analysis (39). Among all of these fields, deep learning showed advantages over many previous related technologies (40, 41). For example, in lung cancer research, deep learning methods can be used to identify biomarker genes on pathological images (42, 43). The success of Residual Networks in the ImageNet Large Scale Visual Recognition Competition in 2015 brought the ResNet model into the limelight. When ResNet18 predicted MSI on H&E tissue section images of gastric adenocarcinoma (STAD) and CRC, it not only had a shorter training time but also achieved an AUC of 0.84 (44). Moreover, it has been reported that ResNet50 has demonstrated exciting performance results in breast cancer and skin cancer classification (45, 46). ResNet18 includes convolution layers and fully connected layers. Compared with ResNet50, ResNet18 lacks the reduction of the corresponding dimension and the function given by the batch norm (BN) layer, which may be the reason why the performance of ResNet18 in predicting the TMB is slightly lower than that of ResNet50 (47). ResNet34, VGG16, and AlexNet can be regarded as the classic models in the deep CNN. After AlexNet was proposed in 2012, it has triggered a boom in its application and plays an important role in the research of medical images. Notably, the structure of VGG16 is very simple. However, the number of VGG network channels is too large, and its structure determines that it requires more parameters and brings more memory usage (48). In addition, the VGG16 network structure is too densely connected, resulting in a long training time, these factors may lead to the relatively poor effect of VGG16 in this study. Currently, the research of deep learning on medical images is quite mature, and most of its achievements have also been recognized and practically applied in clinical practice.

In our research, we used deep learning to identify and analyze CRC histopathological images and achieved the purpose of predicting the TMB. However, the content of the TMB in tumors was seldom estimated using deep learning previously. The reason may be the difference between gene level and phenotype level. DeepHE divides the complete WSI into 512 × 512 H&E slices and predicts the TMB probability. Our results, shown in **Figure 2**, illustrate the promise of exploring associations between cancer genotypes and phenotypes.

To save the detection time of the TMB, researchers have also explored single-panel sequencing methods and more clinically practical panel-based methods. However, many factors like the protein-coding regions of the panel, the association of selected genes with tumors, and the number of genes will affect the ACC of the results (49). DeepHE extracts the potential features of TMB on the pathological images of tumors through deep learning, which does not depend on the selection of genes. Therefore, these shortcomings of the gene panel do not exist for the DeepHE method.

Although DeepHE showed good performance, it was still controversial in many aspects. Firstly, our samples have been delineated and annotated by professional pathologists, and the pathological images have been segmented and screened. The addition of professional pathologists makes this study more professional in medicine and more promising for clinical use. These steps are not required by some TMB detection methods, but those methods still achieved good results. Second, our data all came from FFPE images in TCGA, and no independent validation set was established. Moreover, much complex information and noise in these images cannot be completely analyzed and removed that could affect the ACC and persuasiveness of DeepHE.

## Materials and Methods

### Data Sources

TCGA (https://www.cancer.gov/tcga) is a joint cancer multi-omics analysis database co-founded by the National Cancer Institute and the National Human Genome Research Institute in 2006. From TCGA database, we have collected all available FFPE images of CRC patients; this type of images is often used for clinical diagnostic analysis and is stored simply. This manner does not affect the pathology contained in FFPE tissue, thus guaranteeing the ACC of our model (50). The data resolution was chosen as ×40 and submitted to professional pathologists in SVS format. The TMB distribution of patients is known and published in TCGA, and 20 or 10 were used as the cut points to categorize patients. Patients whose TMB content is higher than the threshold were marked as 1 and recorded as TMB-H, while those lower than the threshold are marked as 0 and recorded as TMB-L.

### Data Processing

In this study, CRC images were downloaded from TCGA and were classified by patients. There are 611 sets of image data in total. According to the diagnosis of pathologists, 6 groups of data have unclear tumor areas in the images, and 96 patients were excluded because the TMB information is missing. WSIs of 509 CRC patients were used for model training in this study. There are thousands of pixels in a WSI, which often contains too much complex information and is not conducive to the analysis of the features on these images by DeepHE. The DeepHE model divided the patient’s WSI scan into H&E slices of 512 × 512 pixels. These slices were used for subsequent training, as shown in **Figure 1C**.

**Figure 4:**
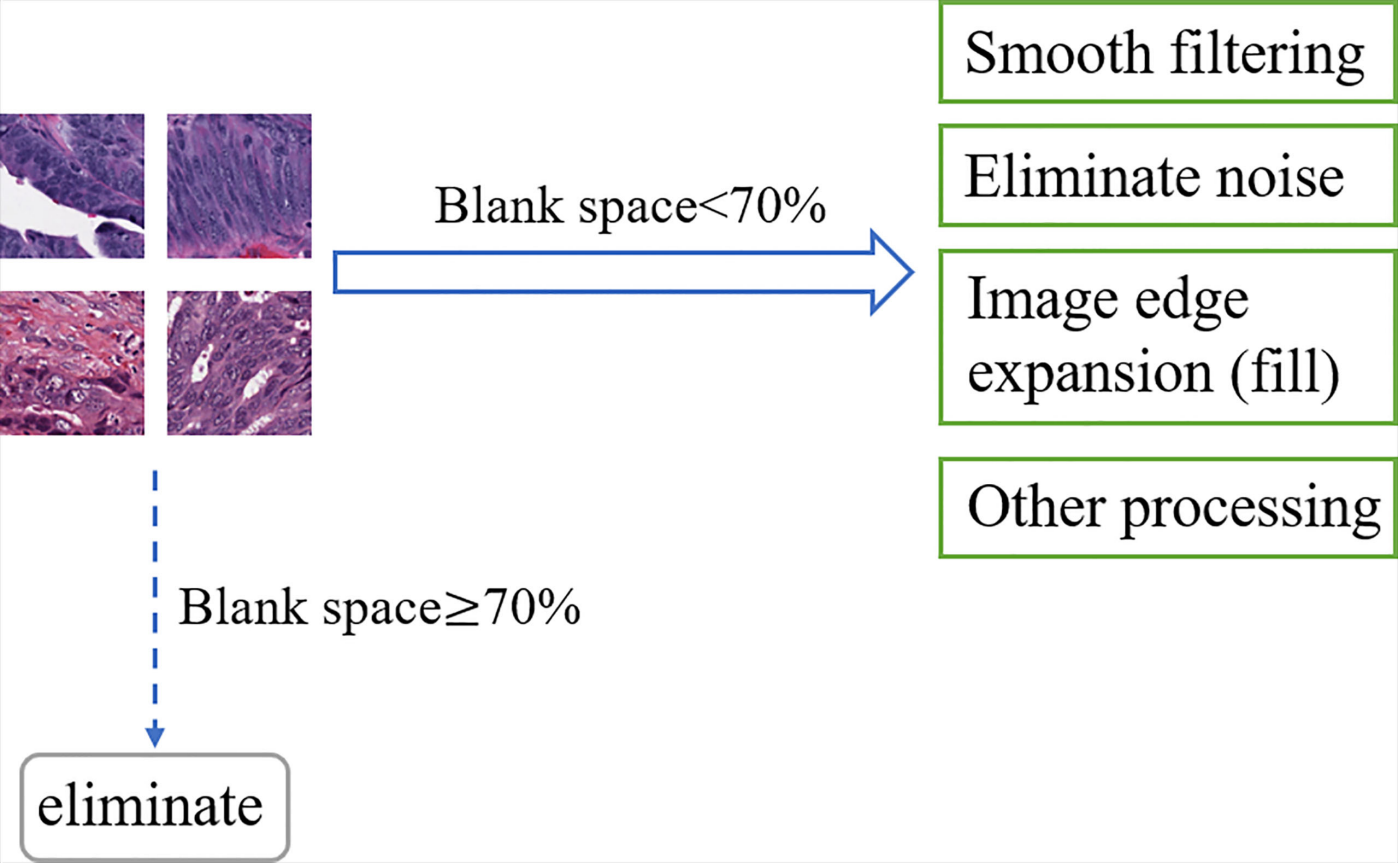
OpenCV process.

Noise information, blurred areas, and blank areas in H&E slices have a non-negligible impact on model training, such as false-positive results and capture feature deviations; then, that information must be paid attention to. As an open-source computer vision library for image processing, OpenCV has powerful and reliable image processing capabilities to reduce the research cost and time of researchers (51). Based on OpenCV, this research regarded H&E slices as pixel matrices and performs segmentation, signature detection, and noise removal for specified targets. After reading the slice data, OpenCV was used to calculate the ratio of the number of blank area pixels in the slice to the slice area, and 70% was used as the threshold to screen samples suitable for predicting target genes. Denote *K*_0_ as the real value of the pixel in the image, and the noise pixel as *L*, then *K* = *K*_0_ + *L*. OpenCV collected many pixels in the image and calculated the average value to make the value of *L* tend to 0, then used the average values to represent the new pixel values that achieve smooth filtering and noise elimination. In addition, OpenCV also shows the functions of image edge expansion (filling), highlighting important parts of the image and adjusting brightness to improve image quality, as shown in **Figure 4**.

In H&E slices of CRC, some images inevitably contain more microvessels, inflammatory cells, microfibrils in the background, and are wrinkled and blurry. Once these images were identified, they were abandoned. After a series of program operations and careful screening, a total of 1,586,826 CRC H&E slices remained. The high quality of these slices ensures the ACC and reliability of the results of the TMB prediction model.

**Figure 5:**
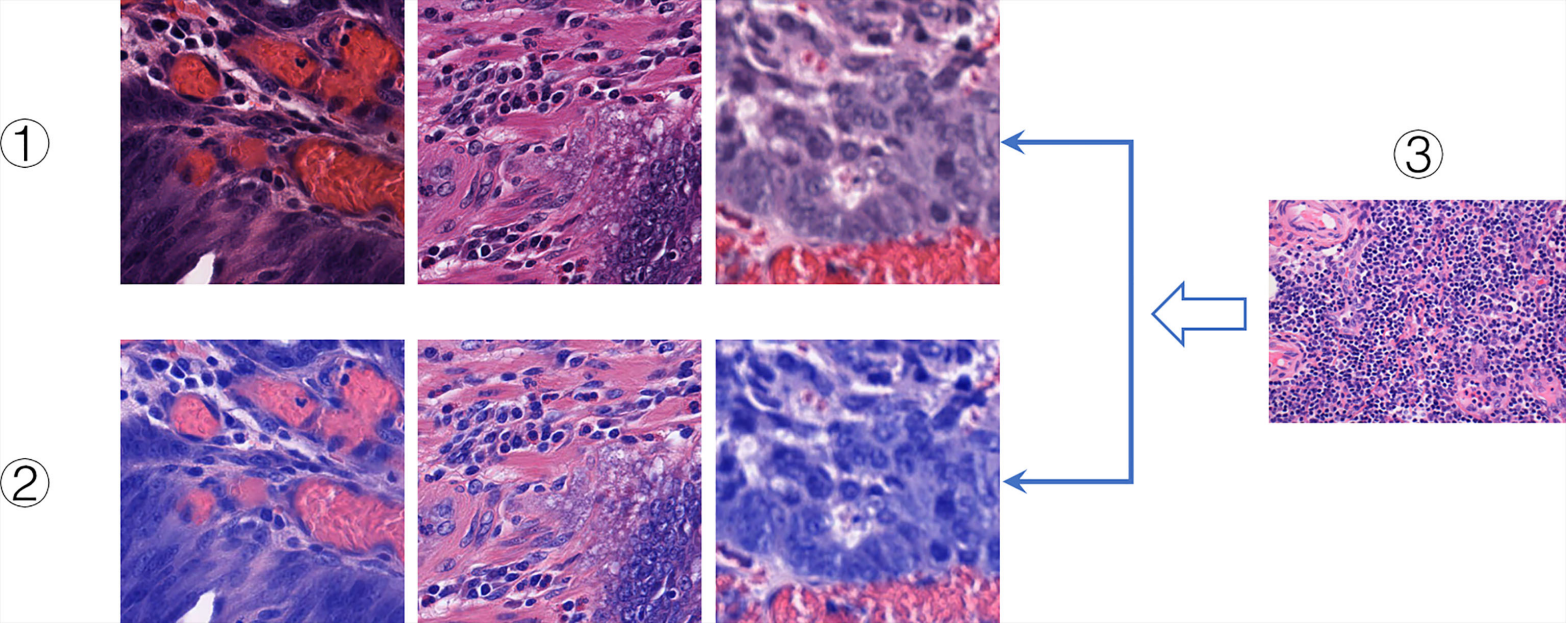
Color normalization. I. Original slice images. II. Color-normalized slice images. III. Reference images.

Hematoxylin and eosin staining are commonly used staining methods in FFPE sectioning technology. Hematoxylin can differentiate cell structures into various colors, while eosin stains the cytoplasm and intercellular substance. There are often differences in the color of each structure. And there are many reasons for color differences, including temperature, solution dose, tissue or cell type, changes in cell cycle, and histopathology (52). Therefore, in the study, H&E slices often show different colors on the same structure, which increases the difficulty of DeepHE in capturing the target information during the training process. To address this issue, we incorporated a color normalization method into the development of the DeepHE model. The color normalization employs an unsupervised deep convolutional Gaussian mixture model (DCGMM) to identify color information in H&E tissue slice images and converts them into a reference image, as shown in **Figure 5**. The color normalization method only transforms the chromaticity of H&E images; the spatial structure and pathological information on it do not change. This method does not require labels and premise assumptions; it also has the capability of automatic learning (53).

The Gaussian mixture model (GMM) can be regarded as a linear group sum of multiple Gaussian functions. Using GMM, the number of clusters n can be specified, and the random variable data are *f_n_*. The training of GMM is to cluster points according to the distance between two different pixels to maximize the expectation:


(4.1) $$\beta (n)=\sqrt{{\left(f_{n}-\theta_{n}\right)}^{T}\sum_{n}^{-1}\left(f_{n}-\theta_{n}\right)}$$

where *θ_n_* represents the mean, and the process is very similar to the k-means algorithm.

When the normal distribution is ∂ , the n-nt data are $\partial \left(f_{n}|\theta_{n},\gamma_{n}\right)$ , and GMM satisfies:


(4.2) $$\alpha \left(n\right)=\sum_{n=1}^{N}\lambda_{n}\partial \left(f_{n}|\theta_{n},\gamma_{n}\right)$$

where *γ_n_* is the covariance matrix $\sum_{n=1}^{N}\lambda_{n}$ is the weight of $\partial \left(f_{n}|\theta_{n},\gamma_{n}\right)$

which satisfies the condition $\sum_{n=1}^{N}W_{n}=1$ (54).

The DCGMM model is a probability distribution model based on GMM. The model is generated by linear superposition of an *N*-dimensional GMM. When *λ_n_* is the prior condition of *f_n_* and the data are *n*, it satisfies:


(4.3) $$P(n)=\frac{\lambda_{n}\partial \left(f_{n}|\theta_{n},\gamma_{n}\right)}{\sum_{i=1}^{N}\lambda_{j}\partial \left(f_{n}|\theta_{n},\gamma_{n}\right)}$$

When *f_n_* is the submodel data, all submodels can finally form a DCGMM model whose (natural) log-likelihood function is:


(4.4) $$\ln P\left(x_{k}|\left(\lambda_{n},\theta_{n},\gamma_{n}\right)\right)=\sum_{k=1}^{K}\ln P\left(x\right)$$

At this time, the selection and change of the parameter (*λ_n_*, *θ_n_*, *γ_n_*) have a decisive effect on the effect of the DCGMM model.

### Deep Learning Algorithms

**Figure 6:**
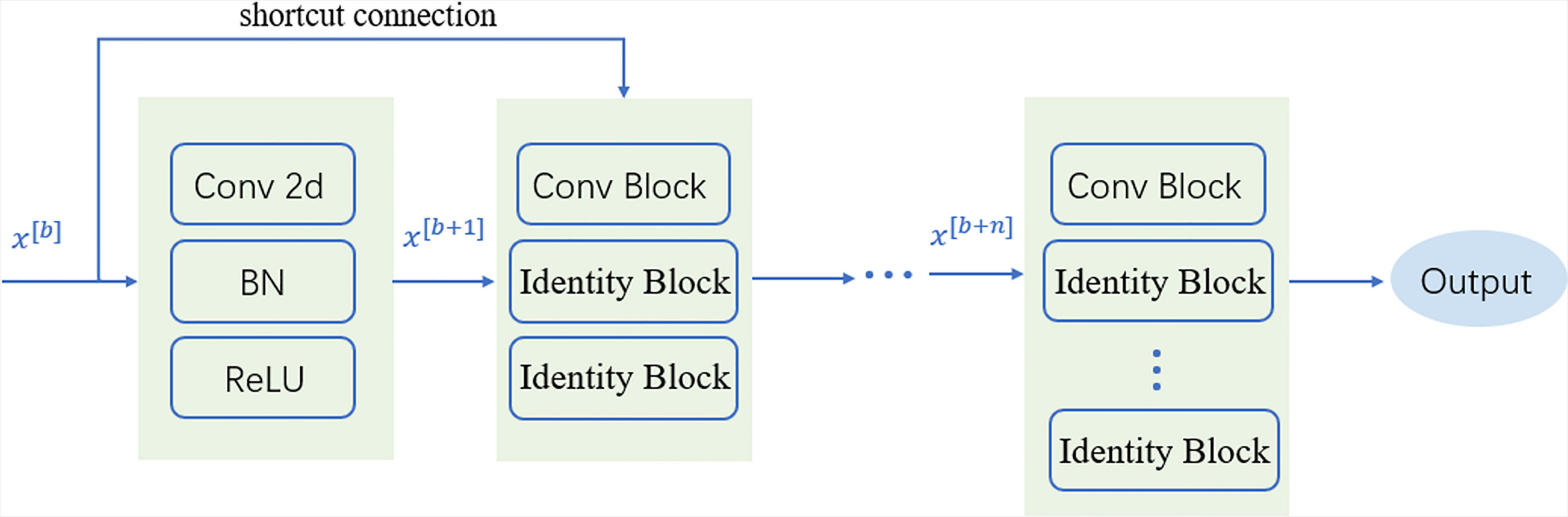
Residual network.

ResNet50 is one of the methods in ResNet. It contains two basic blocks, Conv Block and Identity Block. Usually, Bottlenecks is included in the four blocks, and the number of channels is reduced by a 1 × 1 convolutional layer to half, followed by 3 × 3 and a 1 × 1 convolution to achieve dimensionality reduction and pooling of these images, reducing the amount of subsequent computation and outputs to the next block. Identity Block does not change the dimension of the data itself; it performs the mapping of the data itself. As a result, the network can be extended to a deeper level, which will make the model feature extraction better and improve the model’s classification ACC of image features. The nature of the residual block skip link reduces the training time, which is shown in **Figure 6**. After the data are output, they can linearly reach the input layer of the following block through the skip link, so that the network only needs to learn the differential information between the input layer and the output layer. Compared with traditional CNN, the introduction of ResNet reduces the loss of information and optimizes the model generalization ability and training speed (55).

In this study, ResNet50 is mainly used to form the neural network part of the DeepHE model, and Conv is used as the convolution layer. After the image data x is input, it will first enter the convolution layer with 32n×n kernels and perform feature extraction by weight. Conv Block will change the network dimensions, so they cannot be directly connected in series. Then, the BN layer is added to normalize the Conv results, smooth the landscape of the entire loss function, and improve the feature extraction accuracy and generalization ability of the network (56). We choose ReLU as the activation function, which can be regarded as an identity mapping model for forwarding calculation. This makes the network sparse and at the same time acts as a regularization to realize the repeated comparison of extracted information and feature confirmation.

## Data Availability Statement

The original contributions presented in the study are included in the article/**Supplementary Material**. Further inquiries can be directed to the corresponding author.

## Author Contributions

WG provided the idea of the thesis. YL and KH performed the study and experiments. YW supervised the experiments. YY and WG wrote the article. YL modified and reviewed the article. All authors contributed to the interpretation of data and to the writing and revision of the article. All authors contributed to the article and approved the submitted version.

## Funding

The study was funded by the Joint Funds for the Innovation of Science and Technology, Fujian province (Grant No. 2019Y9038).

## Conflict of Interest

KH and YW are employed by Genies (Beijing) Co., Ltd.

The remaining authors declare that the research was conducted in the absence of any commercial or financial relationships that could be construed as a potential conflict of interest.

## Publisher’s Note

All claims expressed in this article are solely those of the authors and do not necessarily represent those of their affiliated organizations, or those of the publisher, the editors and the reviewers. Any product that may be evaluated in this article, or claim that may be made by its manufacturer, is not guaranteed or endorsed by the publisher.
