# Supplementary figures and images for: Prediction of Tumor Mutation Load in Colorectal Cancer Histopathological Images Based on Deep Learning
Source: Front Oncol. 2022 May 24;12:906888. doi: 10.3389/fonc.2022.906888 (PMC9171017; doi:10.3389/fonc.2022.906888)

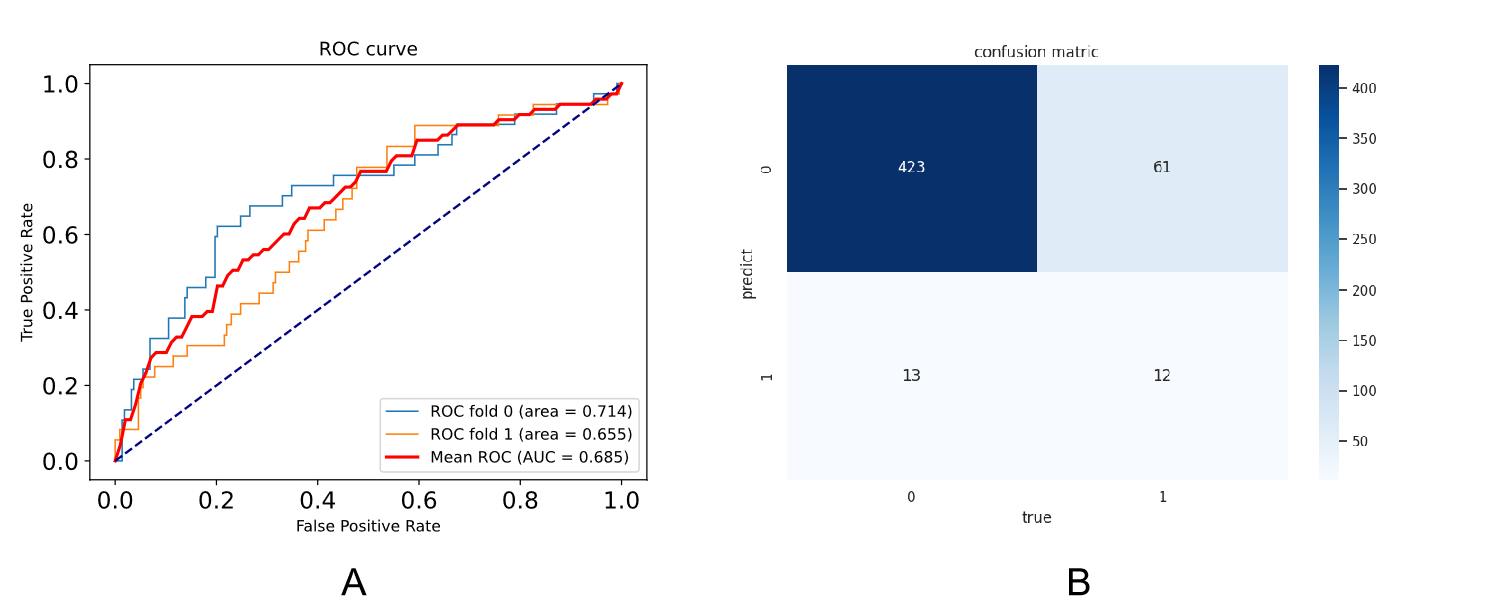

Supplement: Supplementary file 1 [file Image_1.tif]
